# One-step synthesized antimicrobial peptide-functionalized gold nanoclusters for selective imaging and killing of pathogenic bacteria

**DOI:** 10.3389/fmicb.2022.1003359

**Published:** 2022-10-10

**Authors:** Yunqiu Shen, Chaochuan Zheng, Qiaoli Wu, Qilong Wu, Ming Jin, Yayun Jiang, Fuyuan Huang, Yongliang Lou, Laibao Zheng

**Affiliations:** Wenzhou Key Laboratory of Sanitary Microbiology, Key Laboratory of Laboratory Medicine, Ministry of Education, School of Laboratory Medicine and Life Science, Wenzhou Medical University, Wenzhou, China

**Keywords:** nanomaterials, gold nanocluster, antimicrobial peptide, bacterial infection, selective imaging, bacteria killing

## Abstract

The development of multifunctional nanomaterials with bacterial imaging and killing activities is of great importance for the rapid diagnosis and timely treatment of bacterial infections. Herein, peptide-functionalized gold nanoclusters (CWR11-AuNCs) with high-intensity red fluorescence were successfully synthesized *via* a one-step method using CWR11 as a template and by optimizing the ratio of CWR11 to HAuCl4, reaction time, pH, and temperature. The CWR11-AuNCs bound to bacteria and exhibited selective fluorescence microscopy imaging properties, which is expected to provide a feasible method for locating and imaging bacteria in complex *in vivo* environments. In addition, CWR11-AuNCs not only retained the antibacterial and bactericidal activities of CWR11 but also exhibited certain inhibitory or killing effects on gram-negative and gram-positive bacteria and biofilms. The MICs of CWR11-AuNCs against *Escherichia coli* and *Staphylococcus aureus* were 178 and 89 μg/ml, respectively. Surprisingly, cell viability in the CWR11-AuNC-treated group was greater than that in the CWR11-treated group, and the low cytotoxicity exhibited by the CWR11-AuNCs make them more promising for clinical applications.

## Introduction

Infectious diseases caused by pathogenic bacteria pose a growing threat to humans and have caused great economic losses (Cui et al., 2020; Zheng et al., 2022). Traditional bacterial imaging with conventional fluorescent dyes has significant limitations, such as its susceptibility to photobleaching. Antibiotics, on the other hand, were once an effective treatment for such infections, but their inappropriate and excessive use ultimately led to serious antibiotic resistance problems (Llor and Bjerrum, 2014; Pranantyo et al., 2021b). Therefore, there is an urgent need to develop multifunctional nanomaterials with bacterial imaging and killing properties (Wan et al., 2014).

Recently, nanomaterials have been widely used in the fields of catalysis, electronics, and medicine because of their large specific surface area and unique chemical properties (Zhu and Hersam, 2017; Maduraiveeran et al., 2018; Zheng et al., 2018a, 2019a; Wang et al., 2020; Zhou et al., 2022). Gold nanoclusters (AuNCs) are nanomaterials composed of several to hundreds of gold atoms (Matus and Häkkinen, 2021). As a novel fluorescent nanomaterial, AuNCs have the advantages of easy preparation, ultrasmall size, bright fluorescence, high solubility, good biocompatibility, low toxicity, and high stability (Cui et al., 2014; Zhang et al., 2018; Halawa et al., 2020; Geng et al., 2021), which have led to their wide use in the fields of biosensing and biological imaging and treatment (Zheng et al., 2018b, 2019b). Generally, AuNCs are functionalized with different ligands, such as DNA (Wang et al., 2018), proteins (Guo et al., 2020), antibiotics (Yu et al., 2017), and peptides (Zhu et al., 2020). For example, Li and colleagues conjugated vancomycin with water-soluble GSH-coated fluorescent AuNCs to kill *Staphylococcus aureus* (Li et al., 2018). However, the use of antibiotics often leads to the unexpected emergence of resistance (Pranantyo et al., 2021c).

As part of the innate immune systems of advanced living organisms, antimicrobial peptides (AMPs), which are mainly cationic small molecules consisting of 10–50 amino acid residues, have been shown effectively kill bacteria by lysing their membranes(Hancock and Sahl, 2006; Arouri et al., 2009; Pasupuleti et al., 2012; Pranantyo et al., 2019). More importantly, the use of AMPs is less likely to result in resistance than treatment with antibiotics (Jenssen et al., 2006; Pranantyo et al., 2021a). For example, a research team designed a multifunctional antibacterial agent for killing bacteria that combined the photothermal ability of palladium nanoparticles (PdNPs) with the bacterial membrane targeting and lytic activities of AMPs (Andoy et al., 2020). However, this method focuses on only the antimicrobial activity of AMPs. AMPs also are promising recognition elements for bacterial killing and imaging because of their advantages of high stability, capability to be specifically engineered, and high affinity for bacteria (Shirley et al., 2018; Zhou et al., 2018).

Herein, we used the AMP CWR11 (CWFWKWWRRRRR) as a reducing and protecting agent to construct CWR11-AuNCs as a multifunctional material for selective bacterial imaging and killing. The key features of this system are as follows: (i) CWR11 on the CWR11-AuNCs participates in electrostatic and hydrophobic interactions with bacteria but not with macrophage-like cells and (ii) fluorescent AuNCs when combined with aptamers/antibodies can be directly used for bacterial imaging for use in the construction of bacterial-specific detection platforms. Furthermore, we demonstrated that the CWR11 immobilized on the AuNCs retained its antibacterial activity by performing minimum inhibitory concentration (MIC) assays and bactericidal experiments on plates.

## Materials and methods

### Materials and reagents

Chloroauric acid was purchased from Macklin Biochemical Co., Ltd. (Shanghai, China). The CWR11 peptide (CWFWKWWRRRRR) was purchased from China Peptides Co., Ltd. (Shanghai, China). Sodium hydroxide (NaOH) was obtained from Sinopharm Chemical Reagent Co., Ltd. (Shanghai, China). 3-(4,5)-Dimethylthiazol-2,5-diphenyltetrazolium bromide (MTT) was purchased from Beyotime Biotechnology Co., Ltd. (Shanghai, China). Ultrapure water (18.2 MΩ) was used throughout the experiments.

### Synthesis of CWR11-AuNCs and bovine serum albumin-protected AuNCs

CWR11-AuNCs were synthesized according to a previous method (Zhang et al., 2017) with minor modifications. In this work, CWR11-AuNCs were synthesized by mixing CWR11 (0.4 mM) and chloroauric acid (25 mM) in 2.5 ml of water with vigorous stirring. Then, NaOH solution (1 M) was added to the above mixture to adjust the pH to 11. The solution was vigorously stirred for another 30 min at room temperature. Finally, the solution was sealed and placed in the dark for 24 h at 55°C. Then, the mixture was dialyzed against ultrapure water (MWCO 5 kDa) for 3 days to obtain a purified solution of CWR11-AuNCs, which were stored at 4°C for further use.

Bovine serum albumin-AuNCs were synthesized according to a classical experiment (Xie et al., 2009). Briefly, chloroauric acid (10 mM) was added to BSA solution (50 mg/ml) under vigorous stirring. NaOH solution (0.5 ml, 1 M) was introduced 2 min later, and the reaction was allowed to proceed under vigorous stirring at 37°C for 12 h. The obtained solution was stored at 4°C for further use.

### Characterization of the CWR11-AuNCs

The morphology and size of the CWR11-AuNCs were determined by transmission electron microscopy (TEM; JEOL2100F). The zeta potential of the material in aqueous solution at 25°C was measured with a Malvern Nano-ZS90 instrument. The UV–Vis and fluorescence spectra of the CWR11-AuNCs were measured with a Spectra Max M3. The elemental composition of the lyophilized compound was determined by Escalab 250Xi X-ray photoelectron spectroscopy (XPS).

Fluorescence quantum yields (QY) of samples were calculated according to the following expression:


$$\Phi_{S}=\frac{F_{S}}{F_{R}}\times \frac{\left(1-10^{-A_{R}}\right)}{\left(1-10^{-A_{S}}\right)}\times \frac{\eta_{S}^{2}}{\eta_{R}^{2}}\times \Phi_{R}$$

where the subscripts *R* and *S*, respectively, refer to the reference and the sample, Φ is the fluorescence quantum yield and Φ _R_ is equal to 0.54 (quinine Sulfate), F is the integrated fluorescence intensity under fluorescence emission spectrum, A is the absorbance at the excitation wavelength, and η is the refractive index of the solvent.

### Bacterial preparation

The *Escherichia coli* (*E. coli*) ATCC 25922, *Staphylococcus aureus* (*S. aureus*) ATCC 25923, and *Acinetobacter baumannii* (*A. baumannii*) ATCC 19606 used in our experiments were preserved in our laboratory. Bacteria were cultured in LB liquid medium at 37°C for 8–12 h. Then, the cultures were centrifuged at 4,000 rpm for 5 min and washed three times with PBS. Eventually, the bacterial solutions were diluted to different concentrations for testing (10–1.00 × 10^9^ cfu/ml).

### Selective imaging of bacteria against macrophage-like cells

The same concentration of CWR11-AuNCs was incubated with respective RAW264.7 cells (1 × 10^6^ cell/cm^2^), *E. coli* (1 × 10^9^ cfu/ml), or *S. aureus* (1 × 10^9^ cfu/ml) in 1.5 ml of PBS at 37°C. Bacterial pellets were collected by centrifugation at 4,000 rpm for 5 min while cells were centrifuged at 1,500 rpm for 5 min. Then, the pellets and cells were washed three times with PBS and resuspended. Ten microliters of the preprepared suspensions were added to clean glass slides that were lightly covered with another glass slide for immobilization. The specimens were examined under an upright fluorescence microscope with a 100× oil immersion lens.

### Antibacterial assays with CWR11-AuNCs

First, 100 μl of CWR11-AuNC solution was added to an *E. coli* or *S. aureus* suspension (1 × 10^5^ cfu/ml). After 1 h of incubation, 10 μl of bacterial suspension was inoculated on an LB solid agar plate and placed at 37°C for 24 h before the number of colonies was counted. In addition, different concentrations of CWR11-AuNCs were added to the bacterial suspensions (McFarland turbidity of 0.5) and placed at 37°C for 18–24 h. Bacterial viability was determined by measuring the absorbance at 600 nm.

### Determination of the CWR11-AuNC minimum biofilm inhibitory concentration and minimum biofilm eradication concentration

*Staphylococcus aureus* and *A. baumannii* were used as typical gram-positive and gram-negative strains, respectively, in this experiment. First, 100 μl of 2 × 10^7^ cfu/ml *S. aureus or* a 10-fold dilution of *A. baumannii* was added to a 96-well plate, and then serial dilutions of CWR11-AuNCs were added for incubation at 37°C for 24 h, using bacterial suspension without CWR11-AuNCs as a control for bacterial growth. The minimum biofilm inhibitory concentration (MBIC) value of the CWR11-AuNCs was determined by crystal violet staining. The minimum biofilm eradication concentration (MBEC) was determined by adding 200 μl of 1 × 10^7^ *S. aureus* and a 20-fold dilution of *A. baumannii* (OD_600_ = 0.2) placed in a 96-well plate at 37°C for 24 h to form biofilms. The medium was then removed and the biofilms were washed gently three times with PBS. Serial 2-fold dilutions of CWR11-AuNCs were added for an additional 24 h of incubation. The lowest concentration of CWR11-AuNCs that produced the crystal violet dye with a color similar to that of the negative control group was taken as the MBEC value.

### Cytotoxicity test

Fetal Human Colon (FHC) cultured in Roswell Park Memorial Institute 1640 medium and 1% streptomycin–penicillin were selected to test the cytotoxicity of the CWR11-AuNCs. First, 100 μl of cells (1 × 10^5^ cell/ml) was added to 96-well plate. After being incubated overnight, different concentrations of CWR11-AuNCs were added to cells. The plates were then placed in a humidified incubator at 37°C containing 5% CO_2_. After 24 h, the solution was removed, and 100 μl of MTT (0.5 mg/ml) was added to each well. After 4 h of incubation, 100 μl of dimethyl sulfoxide (DMSO) was added, and the absorbance at 570 nm was measured.

## Results and discussion

### Schematic diagram of the preparation and applications of CWR11-AuNCs

**Figure 1:**
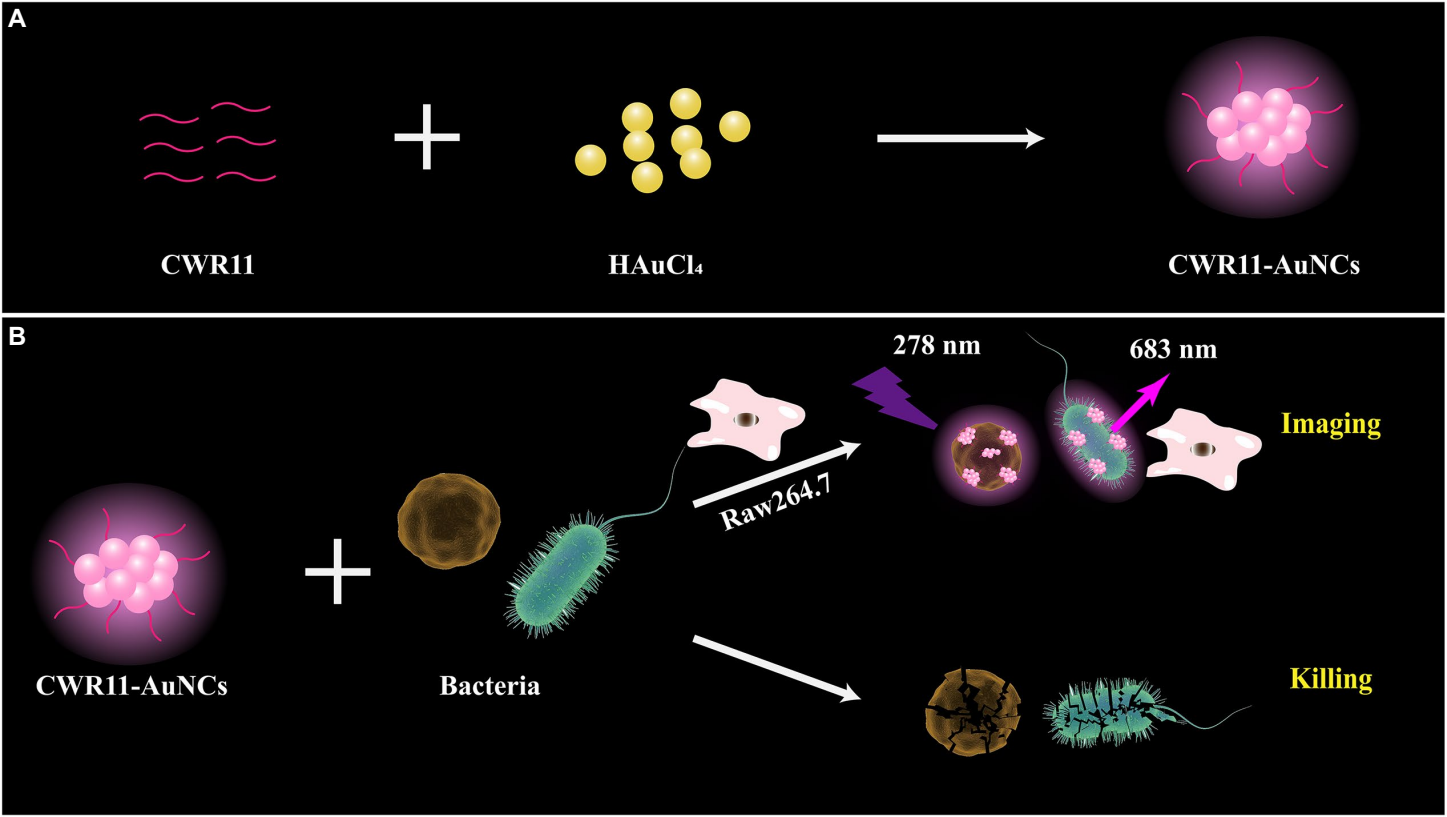
Schematic illustration of the one-pot synthesis of CWR11-gold nanoclusters (AuNCs; **A**) and the use of CWR11-AuNCs to selectively image and kill bacteria **(B)**.

Herein, we prepared multifunctional CWR11-AuNCs with the ability to image and kill bacteria (Figure 1). As shown in Figure 1A, we successfully synthesized CWR11-AuNCs in one step using CWR11 as a protective and reducing agent by forming an Au-S bond between the -SH group of the cysteine (C) residue (Yuan et al., 2016) designed at the end of CWR11 and Au. Figure 1B illustrates the application of CWR11-AuNCs for selective bacterial imaging and killing. The positive charge on the CWR11-AuNCs and the bacterial membrane targeting ability of CWR11 promote the binding of the CWR11-AuNCs to bacteria. Combined with the fluorescence characteristics of CWR11-AuNCs, selective imaging of bacteria can be achieved. In addition, the four tryptophan residues present in CWR11 can be used for membrane insertion and successful bacterial killing.

### Preparation and characterization of the CWR11-AuNCs

**Figure 2:**
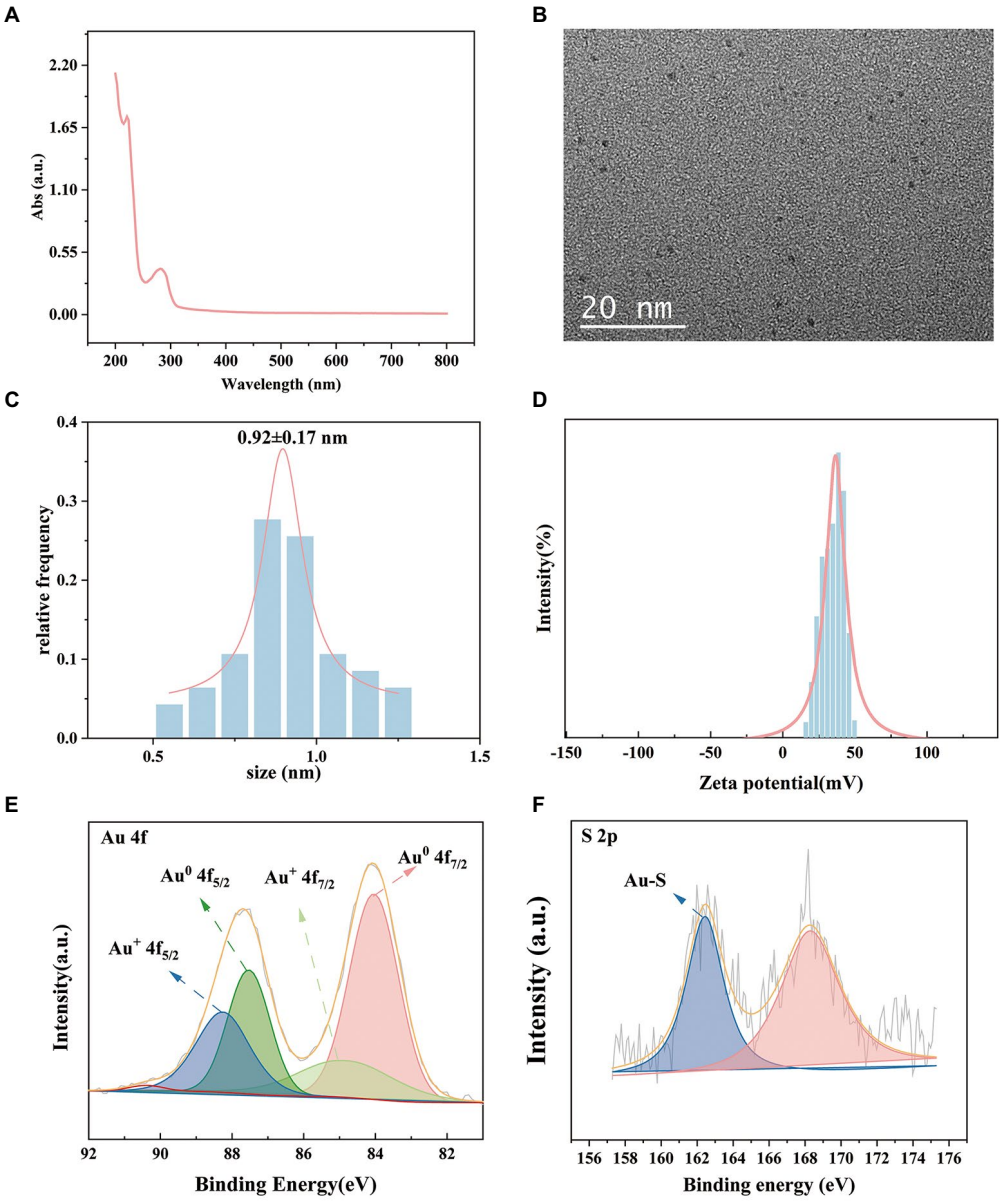
Characterization of the CWR11-AuNCs. UV–vis absorption spectrum **(A)** TEM image; **(B)** size distribution; **(C)** zeta potential; **(D)** Au 4f spectrum; **(E)** and S 2p spectrum **(F)** of the CWR11-AuNCs.

**Figure 3:**
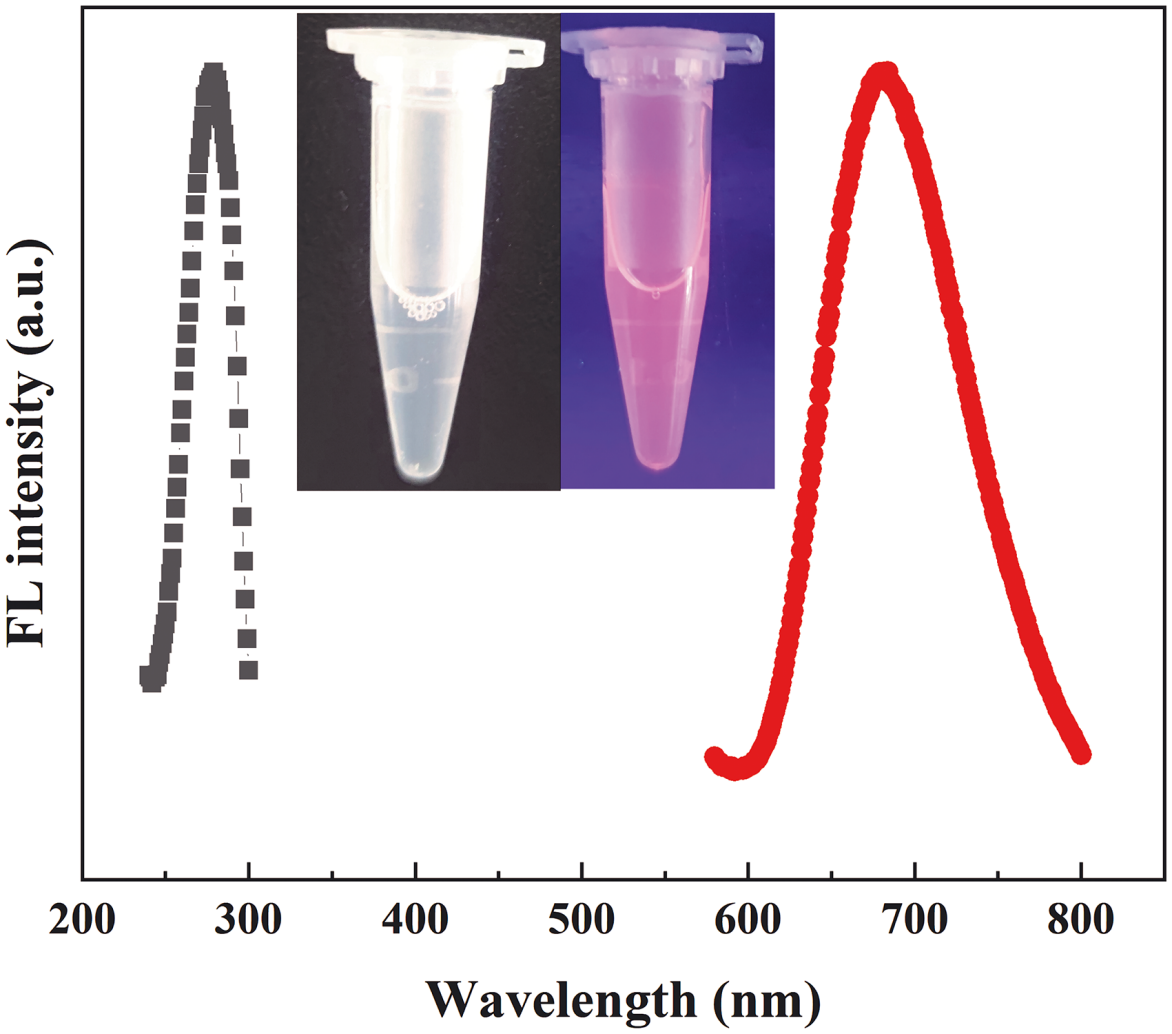
Fluorescence excitation and emission spectra of the CWR11-AuNCs (the inset is photographs of the CWR11-AuNCs under visible and UV light).

We investigated the effects of the ratio of CWR11 to HAuCl_4_, reaction temperature, pH, and reaction time on the preparation of the NCs. Supplementary Figure S1 shows that when the ratio of CWR11 to HAuCl_4_ was 2:1, the reaction temperature was 55°C, the pH was 11, and the time was 24 h, the fluorescence intensity was the strongest. Therefore, the above conditions were determined to be optimal for the preparation of CWR11-AuNCs. The UV–vis absorption and fluorescence spectra of the CWR11-AuNCs are shown in Figure 2. There was no obvious absorption peak between 350 and 400 nm or a surface plasmon resonance peak at 520 nm from gold nanoparticles, indicating the formation of the small CWR11-AuNCs (Figure 2A). The TEM image showed that the CWR11-AuNCs were uniformly dispersed and spherical with a diameter of 0.92 ± 0.17 nm (Figures 2B,C), which was consistent with the UV–vis absorption spectroscopy results. As shown in Figure 2D, the zeta potential of the CWR11-AuNCs was positive, approximately 38 mV, and this positive charge characteristic makes the binding of the NCs to the negatively charged bacterial surface easier. The elemental composition and chemical state were further characterized by XPS. The whole XPS spectrum of the CWR11-AuNCs showed the elements C, N, O, and Au (Supplementary Figure S2), and the Au 4f_7/2_ spectra of CWR11-AuNCs (Figure 2E) was deconvolved into two peaks at 84.0 and 85.0 eV. They are the binding energies of Au^0^ and Au^+^ respectively, which resulted in the Au − S bond. The relative amounts of Au^0^ and Au^+^ in CWR11-AuNCs were determined to be 76 and 24%. Similarly, the peak from Figure 2F centered at 162.5 eV originated from the Au − S bond also proving the AuNCs were successfully formed and protected by CWR11. In addition, fluorescence spectroscopic characterization revealed that CWR11-AuNCs displayed strong red fluorescence under UV light irradiation with QY of 32.1% (excitation wavelength of 365 nm), giving emission and excitation peaks at 682 and 279 nm, respectively (Figure 3). Therefore, the above results indicated that the CWR11-AuNCs were successfully synthesized without the addition of other reducing agents. Based on the properties of the -SH group of the cysteine residues that bound to Au, linking a cysteine residue to the end of other AMPs may provide an easy method to prepare AMP-functionalized AuNCs.

### Selective imaging of bacteria against macrophage-like cells

**Figure 4:**
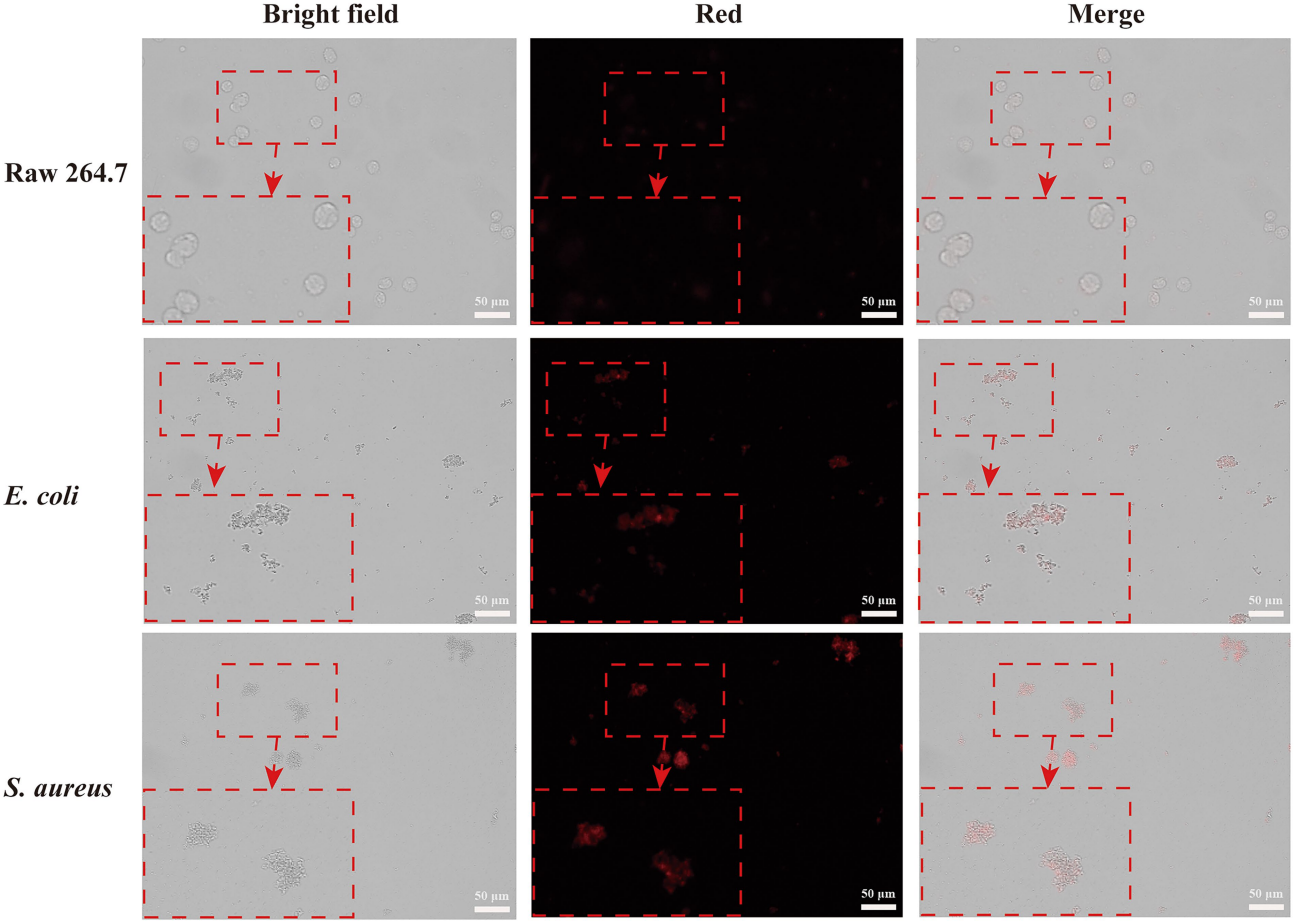
Fluorescence images of RAW264.7 cells, *Escherichia coli* and *Staphylococcus aureus* incubated with CWR11-AuNCs for 15 min, respectively.

To investigate the selective bacteria imaging ability of the CWR11-AuNCs, we chosen the mouse leukemic monocyte macrophage cell line (Raw 264.7) as a control. Specifically, RAW264.7 cells and *S. aureus*/*E. coli* were incubated with CWR11-AuNCs for 15 min. The targeting of the CWR11-AuNCs to these different bacterial species was investigated using fluorescence microscopy. As shown in Figure 4, RAW264.7 cells alone did not show obvious fluorescence after incubation with CWR11-AuNCs, while notable fluorescence was seen after CWR11-AuNC incubation with *S. aureus* and *E. coli*. RAW264.7 cells were also incubated with *S. aureus* or *E. coli* to mimic the macrophage cells infectious tissue environment to further study the selective bacteria imaging ability. These results indicated that CWR11-AuNCs could selectively bind to bacteria in environments where bacteria and cells coexist (Supplementary Figure S3). This opens up the possibility for identifying the location of bacteria in complex environments *in vivo*. We speculated that the CWR11-AuNCs’ selective recognition of bacteria was mainly attributed to the electrostatic interaction between negative-charged bacteria and positive-charged antimicrobial peptide. However, the surfaces of RAW264.7 cells are closer to electroneutrality (Zhu et al., 2011; Qi et al., 2013) making them less susceptible to CWR11-AuNCs binding. To further verify that the bacterial binding properties of the CWR11-AuNCs were attributed to CWR11, bovine serum albumin (BSA)-protected AuNCs were synthesized by the classic way (Xie et al., 2009) as the control. The optical behaviors of the CWR11-AuNCs under different conditions were investigated. As shown in Supplementary Figure S4, the bacteria incubated with the CWR11-AuNCs show clear strong fluorescence, while the bacteria incubated with the BSA-AuNCs show no fluorescence, indicating that the CWR11-AuNCs’ selective recognition of bacteria was mainly attributed to the CWR11.

### Antimicrobial activity of the CWR11-AuNCs

**Figure 5:**
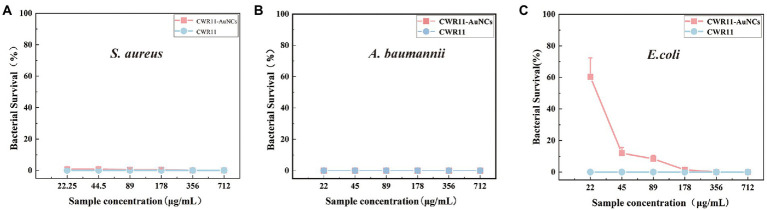
Concentration-dependent bactericidal activities of CWR11 and the CWR11-AuNCs against *Staphylococcus aureus* **(A)**, *Acinetobacter baumannii* **(B)**, and *Escherichia coli* **(C)**.

As mentioned above, CWR11-AuNCs can bind bacteria for selective bacterial imaging. Considering the broad applications of AMPs, we expect this nanomaterial to combine the antibacterial properties of CWR11 with the fluorescent properties of AuNCs. Therefore, the antibacterial activities of different concentrations of CWR11-AuNCs (22, 45, 89, 178, 356, and 712 μg/ml) against *E. coli, A. baumannii*, and *S. aureus*, representing gram-negative and gram-positive bacteria, were tested in plate bactericidal experiments and MIC assays. Both CWR11 and CWR11-AuNCs significantly reduced the activity of *E. coli, A. baumannii*, and *S. aureus* in a dose-dependent manner. Additionally, the results in Figure 5 and Supplementary Figure S5 indicate that the CWR11-AuNCs retained the antimicrobial activity of CWR11. Notably, the CWR11-AuNCs killed almost all *E. coli*, *S. aureus*, and *A. baumannii* at 356, 22, and 22 μg/ml, respectively. The bactericidal effects of low concentrations CWR11-AuNCs against *E. coli* were not as good as those of CWR11, which is consistent with previous reports (Bagheri et al., 2009; Gao et al., 2011). The main reason for the partial loss of CWR11 activity is quite likely due to the physical constraints imposed by the binding of the AuNCs to the bacterial surface; that is, only a small fraction of the CWR11-AuNCs can interact with the bacteria. Thus, reducing the amount of CWR11 could attain bactericidal actions. Moreover, we found that the MICs of the CWR11-AuNCs against *E. coli* and *S. aureus* were 178 and 89 μg/ml, respectively.

### Biofilm inhibitory and eradicating abilities

**Figure 6:**
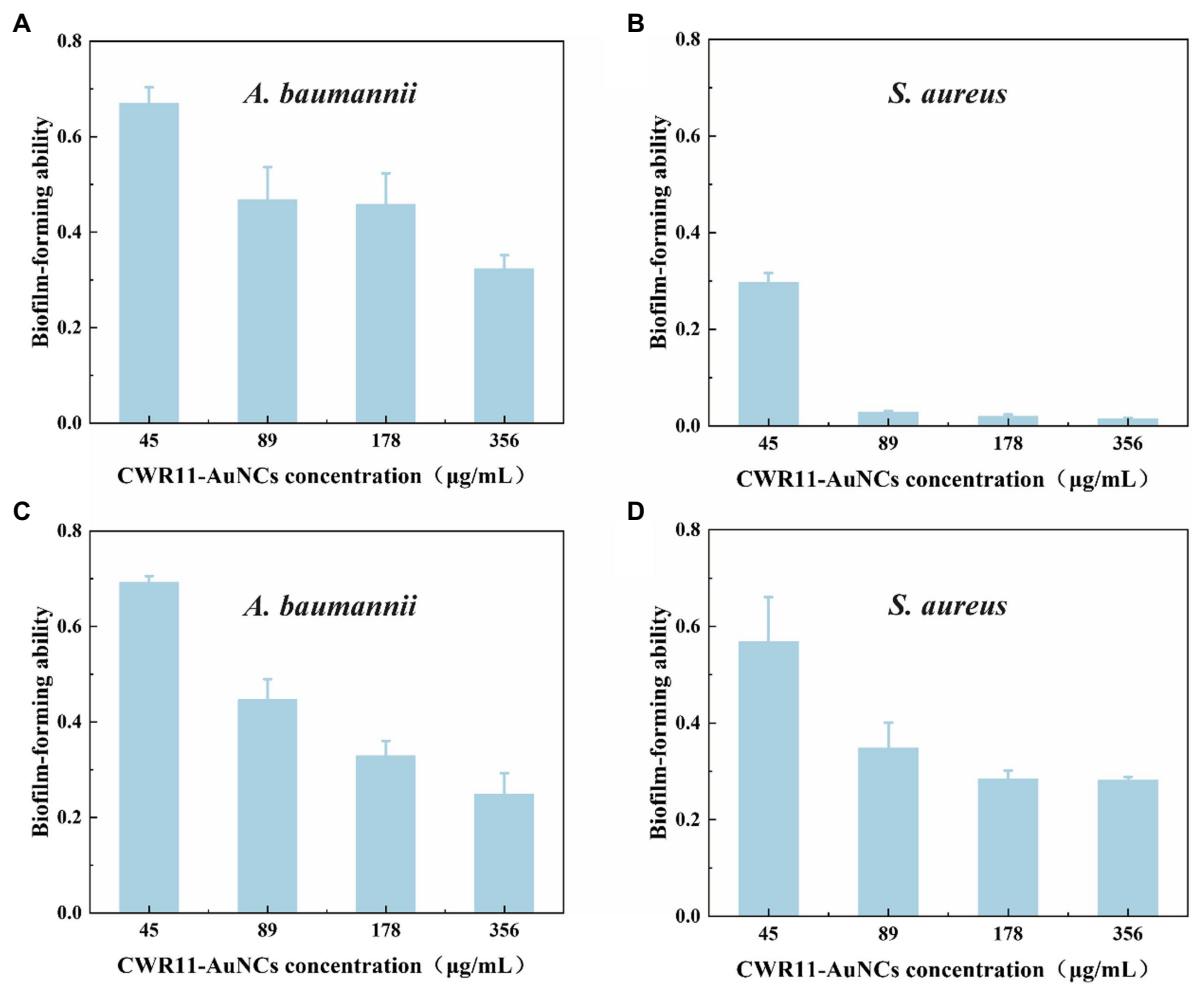
Determination of the ability of CWR11-AuNCs to inhibit or clear biofilms. The membrane survival ability of CWR11-AuNCs (45, 89, 178, and 356 μg/ml) co-incubated with *Acinetobacter baumannii* biofilms and **(A)** *Staphylococcus aureus* biofilms **(B)** for 24 h or their treatment of *A. baumannii* biofilms and **(C)** *S. aureus* biofilms **(D)** for 24 h.

Bacterial biofilms are complexes of microorganisms encased in the extracellular polymeric substance (EPS) matrix that attach to living or abiotic surfaces. Biofilms are generally more difficult to remove than their free-floating counterparts (Costerton, 1999; Costerton et al., 1999). Lank tonic bacteria are more susceptible to antibiotics, the external environment, and the host environment. In contrast, sessile bacteria can resist or evade these destructive factors by forming aggregates, altering their physiology, and exploiting the defects of external factors (La Tourette Prosser et al., 1987; Costerton et al., 1999; Mah and O’Toole, 2001; Spormann et al., 2004). We firstly tested the biofilm survival ability of *E. coli*, *S.aureus*, and *A. baumannii* (Supplementary Figure S6). It showed *S. aureus* and *A. baumannii* are easier than *E. coli* to form biofilm. That means it is infeasible to operate in *E. coli*. Therefore, we chose *S. aureus* and *A. baumannii* rather than *E. coli* to test whether CWR11-AuNCs could affect biofilms. In the minimum biofilm inhibition experiments (Figures 6A,B), taking the value of the biofilm-forming ability without any treatment as 1, *A. baumannii* and *S. aureus* showed different degrees of inhibition after incubation with different concentrations of CWR11-AuNCs. The higher the concentration of the CWR11-AuNCs was, the stronger the inhibition effects were. Additionally, the trend in the minimum biofilm clearance concentration data was consistent with the above results (Figures 6C,D).

### Biocompatibility of the CWR11-AuNCs

**Figure 7:**
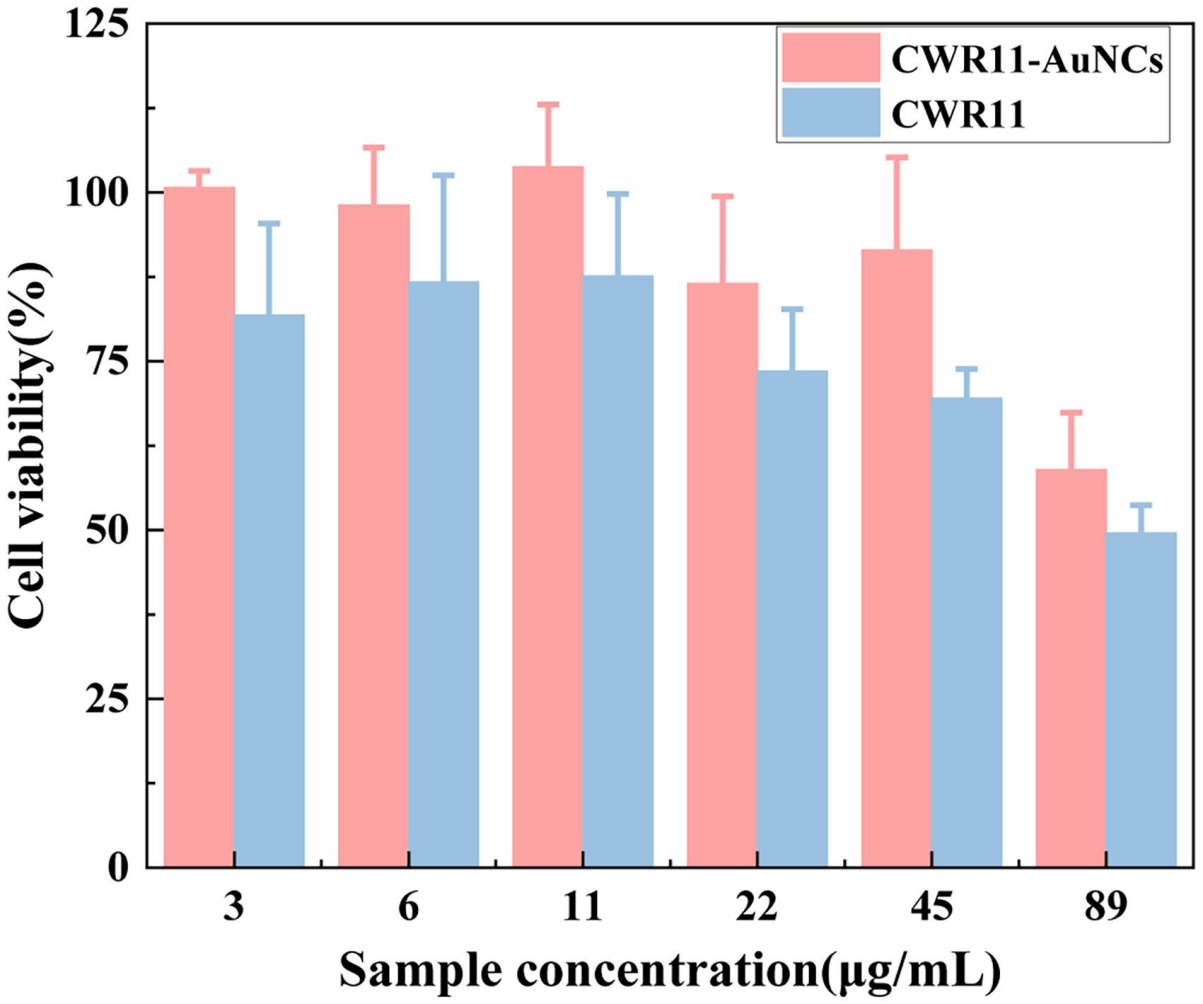
*In vitro* safety assessment. Effects of different concentrations of CWR11-AuNCs on the viability of Fetal Human Colon (FHC) cells.

For all antimicrobial materials to be used *in vivo*, it is imperative to ensure that they are selective, and highly active against bacteria and nontoxic to cells. Here, we assessed and compared the *in vitro* cellular biocompatibility of CWR11 and CWR11-AuNCs using an MTT assay in FHC cells. As shown in Figure 7, compared with CWR11, CWR11-AuNCs were significantly less toxic. When the concentration of the CWR11-AuNCs was 45 μg/ml, the survival rate of HeLa cells still reached 85%, while less than 70% of the FHC cells treated with the same concentration of CWR11 survived. This result indicates that CWR11-AuNCs have relatively good biocompatibility compared with CWR11 alone, which is expected to be advantageous for their use to treat infectious diseases caused by bacteria in the body.

## Conclusion

We successfully synthesized CWR11-AuNCs with high-intensity red fluorescence *via* a one-step synthesis. The synthesized CWR11-AuNCs exhibited the characteristics of bacterial binding and selective imaging in an environment where bacteria and cells coexist, making it possible to determine the location of bacteria in complex *in vivo* environments without interference. Moreover, the bactericidal effect of CWR11-AuNCs was comparable to that of CWR11 to a certain extent, indicating that the antibacterial efficacy of CWR11 was still retained after of its conjugation with the AuNCs. Additionally, the CWR11-AuNCs displayed lower cytotoxicity than CWR11 alone, which is expected favorable for using this nanomaterial to treat infectious diseases caused by bacteria *in vivo*. Thus, the successful application of CWR11-AuNCs for bacterial imaging and antibacterial therapy and their good biocompatibility provide the possibility for their application *in vivo*.

## Data availability statement

The raw data supporting the conclusions of this article will be made available by the authors, without undue reservation.

## Author contributions

CZ and YS: development or design of methodology and writing—original draft preparation. QaW, MJ, YJ, QlW, and FH: investigation, validation, and writing—reviewing and editing. LZ and YL: conceptualization, writing—reviewing and editing, and supervision. All authors contributed to the article and approved the submitted version.

## Funding

This work was supported by Zhejiang Provincial Natural Science Foundation of China under grant no. LQ21H200008, the Science and Technology Bureau of Wenzhou under grant no. Y2020108, National Major Infectious Disease Prevention Projects under grant no. 2018ZX10201001-009, and The Key Discipline of Zhejiang Province in Medical Technology (First Class, Category A).

## Conflict of interest

The authors declare that the research was conducted in the absence of any commercial or financial relationships that could be construed as a potential conflict of interest.

## Publisher’s note

All claims expressed in this article are solely those of the authors and do not necessarily represent those of their affiliated organizations, or those of the publisher, the editors and the reviewers. Any product that may be evaluated in this article, or claim that may be made by its manufacturer, is not guaranteed or endorsed by the publisher.

## Supplementary material

The Supplementary material for this article can be found online at: https://www.frontiersin.org/articles/10.3389/fmicb.2022.1003359/full#supplementary-material
