# Supplementary material for: One-step synthesized antimicrobial peptide-functionalized gold nanoclusters for selective imaging and killing of pathogenic bacteria
Source: Front Microbiol. 2022 Oct 10;13:1003359. doi: 10.3389/fmicb.2022.1003359 (PMC9589054; doi:10.3389/fmicb.2022.1003359)
Supplement: Supplementary file 1 [file Data_Sheet_1.DOCX]

Supplementary Material


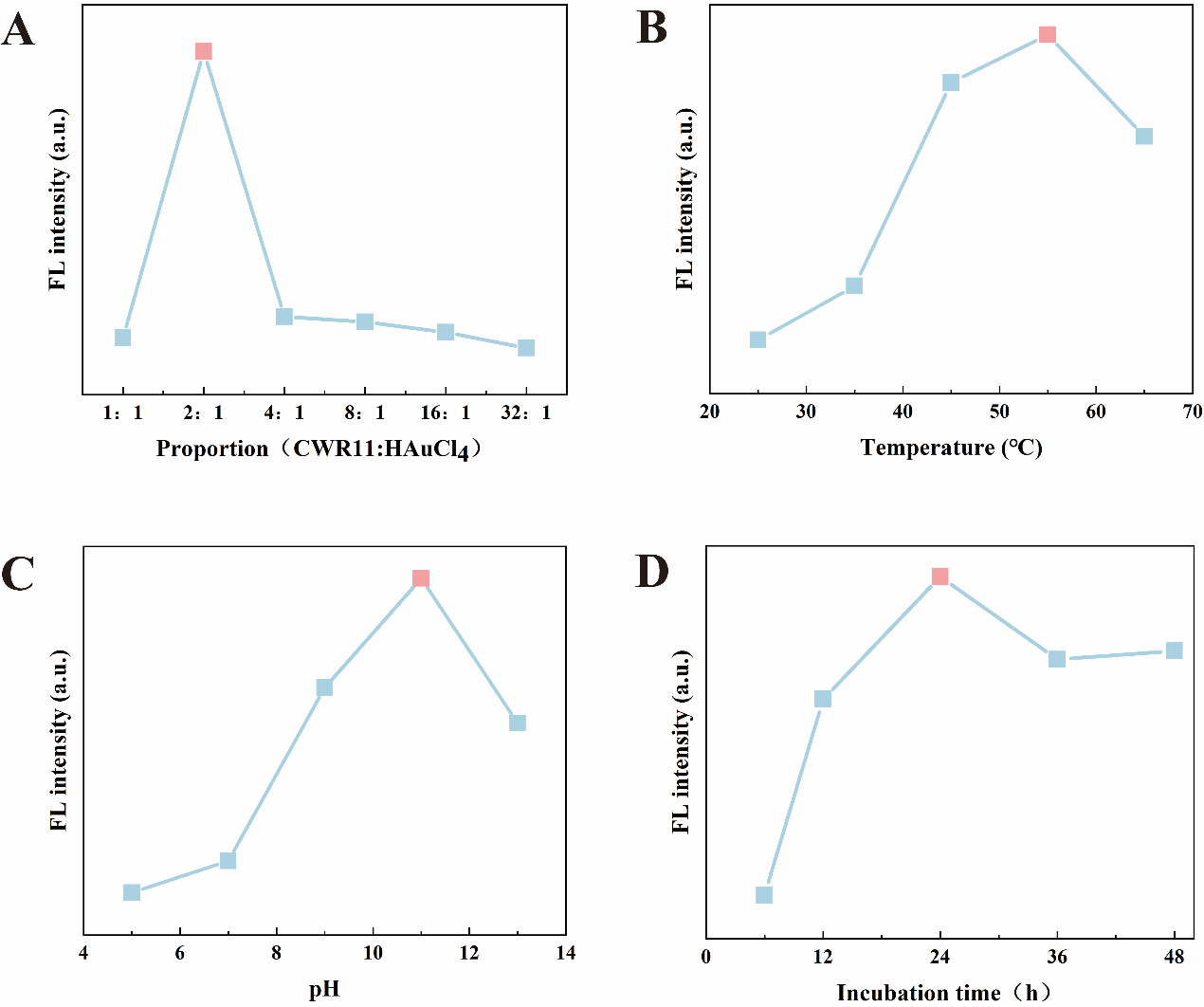


**Figure S1.** Fluorescence spectra of CWR11-AuNCs synthesized using different ratios of CWR11 to HAuCl_4_(A), temperature(B), pH(C) and incubation time(D).

Figure S2. XPS spectrum for Au-NCs


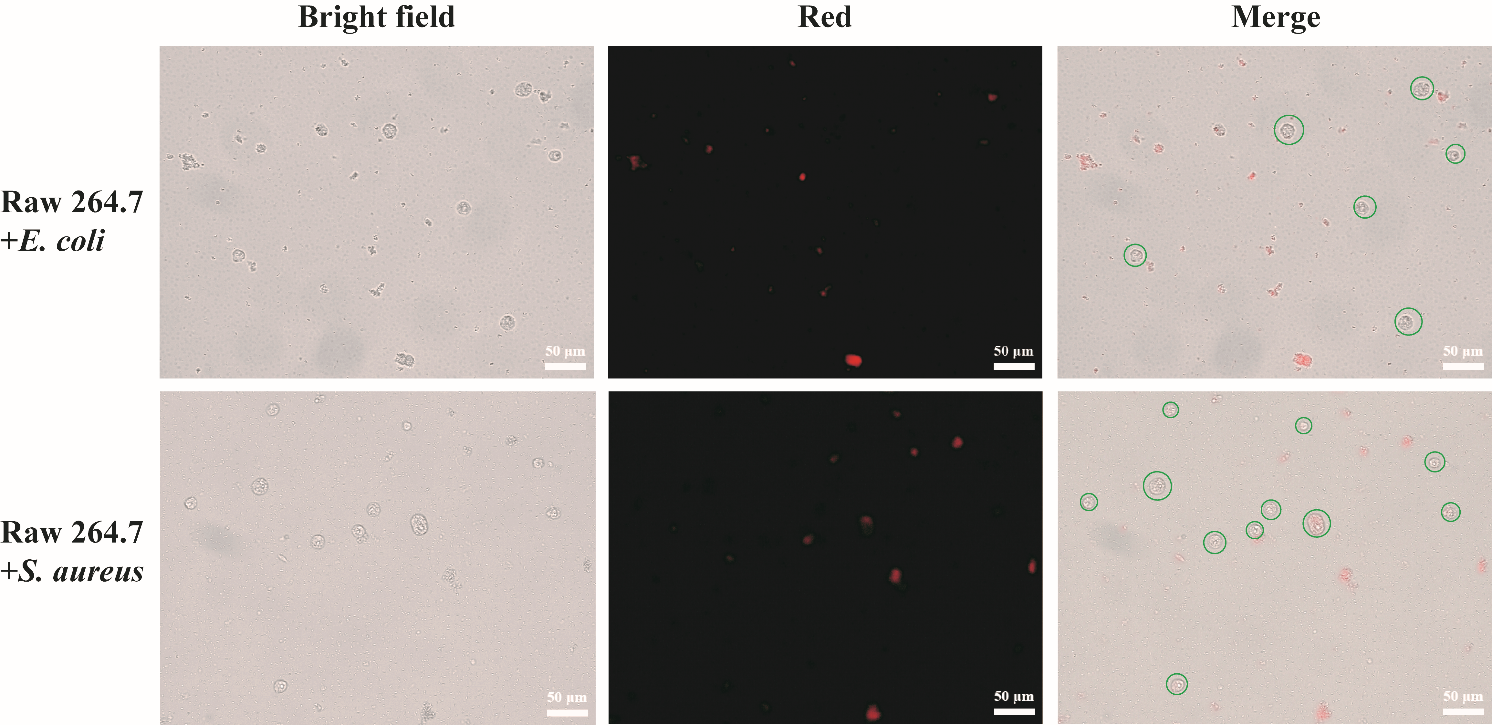


**Figure S3.** Fluorescence images of the mixture of RawRAW264.7 cells and *E. coli* or *S. aureus* incubated with CWR11-AuNCs for 15 min., respectively. Green circles indicate: RawRAW264.7 cells.


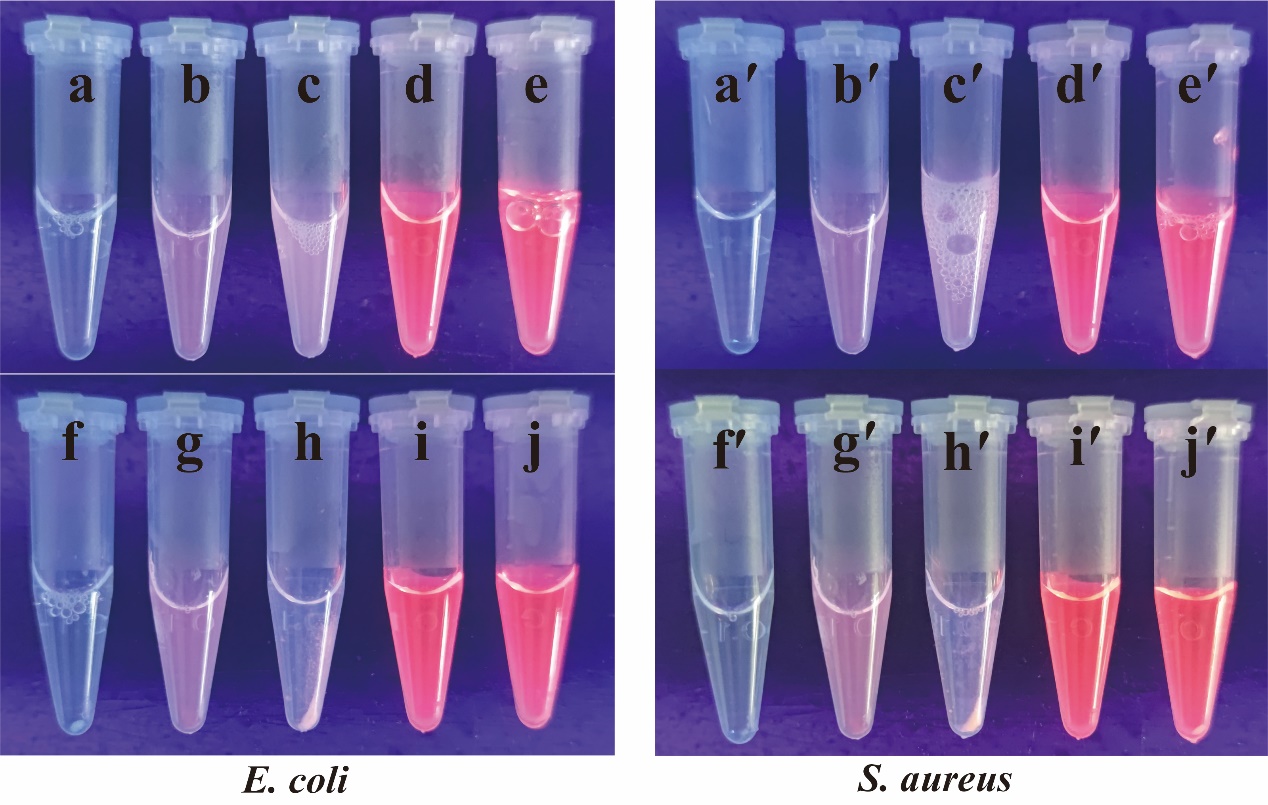


**Figure S4.** Optical photographs of *E. coli* (a and f), CWR11-AuNCs (b and g), CWR11-AuNCs+*E. coli* (c and h), BSA-AuNCs (d and i), BSA-AuNCs+*E. coli* (e and j), *S. aureus* (a′ and f′), CWR11-AuNCs (b′ and g′), CWR11-AuNCs+*S. aureus* (c′ and h′), BSA-AuNCs (d′ and i′), BSA-AuNCs+*S. aureus* (e′ and j′) before (a-e, a′-e′) and after centrifugation (f-g, f′-j′). Excitation: 365 nm. *E. coli*/*S. aureus*: 1×10^9^ cfu/mL.


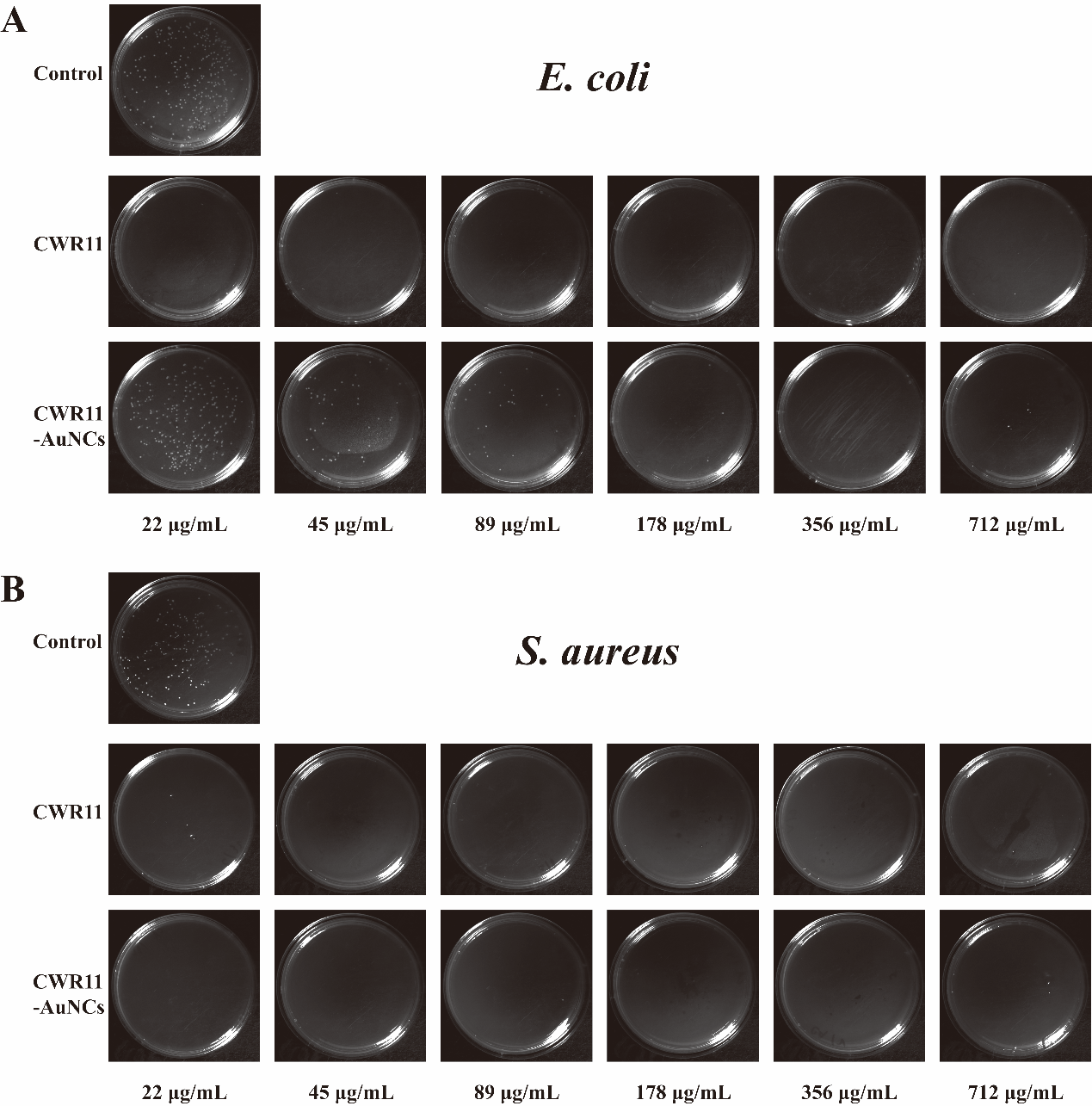


**Figure S5.** Photographs of the agar plates of colonies of *E. coli*(A) and *S. aureus*(B) after treatments of CWR11 or CWR11-AuNCs.





**Figure S6.** Comparison of film-forming ability with different concentrations of E.coli, A.baumannii and S.aureus(the concentrations of Escherichia coli and Staphylococcus aureus were 0.5/1/2×10^7^ cfu/mL, and the concentration of Acinetobacter baumannii was that diluting the basis of OD_600_=0.2 10/20/40 times above)
